# Relationship Between Activity Tracker Metrics and the Physical Activity Index and Their Association With Cardiometabolic Phenotypes, Subclinical Atherosclerosis, and Cardiac Remodeling: Cross-Sectional Study

**DOI:** 10.2196/71213

**Published:** 2025-09-24

**Authors:** Weiting Huang, Mark Kei Fong Wong, Enver De Wei Loh, Tracy Koh, Alex Weixian Tan, Xiayan Shen, Onur Varli, Siew Ching Kong, Calvin Woon Loong Chin, Swee Yaw Tan, Jonathan Jiunn Liang Yap, Eddie Yin Kwee Ng, Khung Keong Yeo

**Affiliations:** 1 National Heart Centre Singapore Singapore Singapore; 2 Nanyang Technological University Singapore Singapore

**Keywords:** digital health, wearables, cardiovascular health, psychometric properties, metabolic health

## Abstract

**Background:**

Consumer wearable technology quantifies physical activity; however, the association between these metrics and cardiometabolic health requires further elucidation.

**Objective:**

This study identified latent factors derived from Fitbit heart rate metrics and their relationship with cross-sectional cardiovascular phenotypes.

**Methods:**

This cross-sectional analysis included 457 participants from the SingHEART study, a multiethnic, population-based study of Asian individuals aged 21 to 69 years recruited in Singapore. Participants wore the Fitbit Charge HR for 7 days, and data on physical activity metrics, self-reported physical activity index (PAI), blood tests, coronary artery calcium scores, and cardiac magnetic resonance imaging were collected. Exploratory factor analysis identified latent factors from Fitbit metrics, and multivariate regression analysis assessed associations with blood and cardiovascular imaging phenotypes.

**Results:**

Higher levels of self-reported PAI were significantly associated with a higher number of calories burned (*P*=.008), number of steps and floors climbed, distance, number of activity calories, and number of very active minutes (*P*<.001). However, there was no association between PAI and other Fitbit metrics. Using exploratory factor analysis, we identified three latent factors measured by Fitbit metrics: (1) elevated metabolic equivalents of task (METs; calories burned per day, minutes per day spent fairly active in 3-6 METs and very active in ≥6 METs, and activity calories), (2) total activity (steps per day, distance in kilometers per day, and number of floors per day), and (3) others, all with a Cronbach α of >0.7. Higher total activity was associated with increased high-density lipoprotein levels (β=0.06; *P*<.001), decreased triglyceride levels (β=−0.10; *P*=.006), and lower BMI (β=−0.63; *P*<.001) after adjustment for age, gender, systolic blood pressure, total cholesterol, and family history of heart disease. The interaction between total activity and elevated METs was associated with lower fasting glucose (β=−0.07; *P*=.004). Elevated METs were associated with higher log(coronary artery calcium+1) and higher BMI (*P*<.001). Total activity was significantly associated with higher indexed biventricular systolic (*P*=.01 for left and *P*=.006 for right) and diastolic volumes (*P*<.001) and higher indexed left ventricular mass (*P*=.005).

**Conclusions:**

We identified 3 groups of wearable metrics with distinct characteristics. While total activity had a significant relationship with self-reported PAI, most metrics of elevated METs did not. Total activity had a consistent and favorable association with lipid and glucose profiles and a dose-dependent association with cardiac remodeling. Elevated METs alone did not appear to have a significant association with favorable cardiovascular profiles. This study suggests that the total activity metrics are robust and dependable when interpreting an individual’s activity levels, with construct validity according to self-reported PAI and a positive association with lipid and glucose profiles, and demonstrate dose-dependent associations with cardiac remodeling after adjustment for demographics and risk factors. Findings related to elevated METs may be due to the Hawthorne effect and require further studies.

## Introduction

### Background

Consumer health wearables are being rapidly adopted by the general population. Surveys have shown that up to 60% of the population own and use wearable devices [1-3]. Physical activity information is the most common measurement provided by current wearable devices. In addition, wearable-reported physical activity data have been studied and validated in various settings to ensure accuracy [4,5]. The abundance of accurate physical activity data presents an opportunity to transform them into actionable information.

Physical activity is a modifiable risk factor toward reducing cardiovascular disease, with up to 30% risk reduction in events among middle-aged adults [6]. Guideline recommendations on physical activity are largely derived from self-reported physical activity in epidemiological studies and its links to cardiovascular risk factors and events [6-13]. Self-reported physical activity has only been moderately correlated at best with actual physical activity [14], suggesting the imprecision of self-reported physical activity. Studies comparing self-reported and wearable-quantified physical activity have mainly looked at their correlation [15,16], but literature on their differences in association with cross-sectional health states is lacking. Less precise self-reported physical activity may mask significant associations with cardiometabolic profiles, which change subtly with physical activity. The inclusion of health wearables in recent large population studies [14,17,18] presents an opportunity to quantify physical activity more accurately. Moreover, there is a strong body of evidence suggesting that interventions using wearable activity trackers result in improvements in the physical activity of users; however, the effectiveness of these interventions on physiological outcomes remains uncertain [19].

### Objectives

Despite widespread interest in the utility of wearable devices in health care, one of the challenges is synthesizing the vast amounts of data collected and integrating them into clinical practice [20]. While most studies have shown that step count consistently shows a positive relationship with cardiovascular health [8,21,22], other metrics such as exercise intensity and sedentary time have been less studied [22]. This study aimed to identify latent factors derived from Fitbit Charge HR (Google) physical activity metrics and correlate them with cross-sectional health outcomes. Considering the differences in activity behavior between weekends and weekdays [7], participants were given the fitness tracker for 1 week to ensure representative physical activity behavior, similar to that in previously conducted wearable studies [18,23].

## Methods

### Study Design

SingHEART (ClinicalTrials.gov NCT02791152) [17] was the first multiethnic prospective population-based study of healthy Asian individuals who completed detailed lifestyle and medical history questionnaires and had their activity levels recorded via wearables and their cross-sectional cardiovascular status detailed via blood tests and imaging (described in the following sections). A total of 457 individuals from SingHEART [17] were included in this cross-sectional study. Healthy male and female participants aged 21 to 69 years without any previous cardiovascular disease (eg, ischemic heart disease, stroke, or peripheral vascular disease) or diabetes mellitus were recruited from the Singapore general public via advertisements (eg, posters and the local newspaper).

### Baseline Blood Tests and Measurements

A comprehensive set of assessments was conducted at baseline. Basic blood tests, including fasting lipids and glucose, and clinical parameters of height, weight, and hip and waist circumference were measured.

### Physical Activity

Participants were given the Fitbit Charge HR wearable device, which tracked heart rate, step count, and intensity of physical activity. The device was worn over a course of 7 days [16]. Step counts were retrieved as daily totals, and the mean number of steps in each day was derived. There were 9 averaged metrics on physical activity presented by Fitbit Charge HR on a per-day basis: calories burned, number of floors, number of steps, distance, minutes spent sedentary, minutes spent lightly active, minutes spent active, minutes spent very active, and activity calories. The various activity intensity levels were previously defined by Fitbit: sedentary, lightly active (1.5-3 metabolic equivalents of task [METs]), fairly active (3-6 METs in at least 10-minute bouts), and very active (≥6 METs or ≥145 steps per minute in at least 10-minute bouts) [24]. Participants were asked to carry on with their regular jobs and physical activity and not to artificially inflate their activity levels so that the wearable was able to capture an accurate representation of their usual lifestyle. Self-reported physical activity was also quantified using the physical activity index (PAI) [25] derived from the General Practice Physical Activity Questionnaire [26].

### Blood Pressure Monitoring

Ambulatory blood pressure (BP) was measured using a cuff monitor (Spacelabs Healthcare models 90227 and 90217A). BP was then analyzed as a continuous variable.

### Coronary Artery Calcium Scoring

All participants underwent noncontrast cardiac computed tomography scans using a 320-slice computed tomography scanner. Coronary artery calcium (CAC) was quantified using the Agatston score [12] via the Vitrea Workstation. CAC was logarithmically transformed into a continuous variable (log[CAC+1]). As this cohort of individuals was free of known cardiovascular disease, the CAC helped detect subclinical, early atherosclerosis.

### Magnetic Resonance Imaging Cardiac Measurements

Cardiovascular magnetic resonance was conducted for all participants using either the Ingenia 3.0T (Philips) or MAGNETOM Aera 1.5T (Siemens Healthineers) scanners. Parameters of cardiac mass, volumes, and function were measured using the cvi42 software (Circle Cardiovascular Imaging) and standardized protocols [27]. Left ventricular mass without papillary muscles was indexed to body surface area according to the DuBois formula, and left ventricular mass index was used for analysis [28].

### Ethical Considerations

This study was conducted in accordance with the Declaration of Helsinki [29]. Written informed consent was obtained from all participants, and the study was approved by the SingHealth Centralised Institutional Review Board (reference 2015/2601). The data used for analysis were anonymized to ensure participants’ privacy and confidentiality. Participants were given SGD $50 (US $39) as compensation for their transport and inconvenience.

### Statistical Analysis: Identifying Latent Factor Measures Using Fitbit Metrics

In this paper, *items* refer to the Fitbit-derived metrics. Using factor analysis, we determined the optimal number of factors using the Kaiser rule (ie, eigenvalue>1). To identify items contributing to factors, we used the criterion of factor loading of >0.4 as a cutoff [30].

Internal consistency reliability (the extent to which the items grouped together are appropriate to measure the same property) was assessed for each factor using the Cronbach α [31]. Construct validity of factors (the ability to measure the intended property in reference to acknowledged standards) was assessed against the self-reported PAI. A value of ≥0.6, 0.4 to 0.5, and 0.2 to 0.3 was considered a strong, moderate, and weak correlation, respectively [32].

Structural equation modeling [33] was used to analyze the relationship between measured items and latent constructs.

After factor analysis using oblique rotation, the identified factors were predicted using regression scoring [34]. Each individual factor was obtained as a weighted sum of standardized versions of the items with a mean of 0.

Intergroup differences in risk factors were evaluated using chi-square tests for categorical variables and ANOVA for continuous variables with Bonferroni correction. Statistical significance was considered as *P*<.05.

Multivariate regression analysis adjusting for age, gender, BMI, 24-hour average systolic BP, total cholesterol, and family history of coronary artery disease testing the identified latent factors was conducted for the lipid and glucose profiles, log(CAC+1), and cardiac magnetic resonance imaging (MRI) variables.

All analyses were conducted using Stata (version 17.0; StataCorp) [35].

## Results

### Overview

The baseline characteristics of the population as a whole and divided by self-reported PAI categories are shown in Table 1. The average age of the participants was 50.05 (SD 9.42) years, and 46.6% (213/457) were male. Most of the participants (234/457, 51.2%) had sedentary jobs, and 4.4% (20/457) had highly active jobs. There was a significantly higher proportion of male individuals in the moderately active and active categories. There was also a significant trend of increasing calories burned, number of steps, distance, number of floors, activity calories, and very active minutes recorded via Fitbit with increasing PAI levels but not for the activity minutes in other MET categories. Significant trends of increasing cardiac remodeling via indexed left and right ventricular volumes and indexed left ventricular mass measured through cardiac MRI with increasing PAI levels were observed. There was no significant association between increasing PAI levels and BP, lipid and glucose profiles, BMI, or log(CAC+1).

**Table 1:** Demographic and clinical characteristics of participants in general and across physical activity index categories (N=457).

			All		Sedentary (n=105)		Moderately inactive (n=106)		Moderately active (n=108)		Active (n=106)		*P* value	
Age (y), mean (SD)			50.05 (9.42)		49.62 (9.86)		51.78 (9.51)		49.31 (9.31)		49.81 (8.57)		.63	
Male sex, n (%)			213 (46.6)		38 (36.2)		38 (35.8)		49 (45.4)		63 (59.4)		<.001	
**Job type, n (%)**														<.001
	Sedentary	234 (51.2)		70 (66.7)		38 (35.8)		68 (63)		54 (50.9)				
	Active	135 (29.5)		0 (0)		60 (56.6)		17 (15.7)		35 (33)				
	Highly active	20 (4.4)		0 (0)		0 (0)		6 (5.6)		5 (4.7)				
Calories burned, mean (SD)			938.74 (353.20)		2065.53 (510.64)		2077.66 (438.31)		2124.99 (451.90)		2264.87 (478.37)		.008	
Number of steps, mean (SD)			938.74 (353.20)		8398.25 (3220.46)		9506.717 (3238.37)		9452.63 (2692.92)		10,525.69 (3591.07)		<.001	
Distance (km), mean (SD)			6.55 (2.35)		5.70 (2.28)		6.50 (2.34)		6.51 (1.95)		7.44 (2.58)		<.001	
Number of floors, mean (SD)			9.03 (7.91)		6.59 (4.59)		9.62 (8.99)		8.65 (6.61)		11.26 (9.74)		<.001	
Sedentary minutes, mean (SD)			873.10 (119.08)		889.45 (110.78)		870.34 (120.07)		859.88 (122.64)		873.14 (122.10)		.34	
Lightly active minutes, mean (SD)			222.40 (64.46)		216.65 (62.20)		231.42 (63.59)		225.05 (65.15)		216.39 (66.49)		.26	
Fairly active minutes, mean (SD)			18.51 (17.43)		15.05 (16.97)		17.76 (15.65)		19.95 (16.80)		21.21 (19.66)		.05	
Very active minutes, mean (SD)			19.11 (19.26)		14.49 (18.04)		17.57 (18.78)		18.35 (14.99)		26.01 (22.83)		<.001	
Activity calories, mean (SD)			931.46 (353.70)		854.76 (378.15)		919.14 (320.72)		934.51 (306.16)		1016.63 (389.61)		.01	
24-h SBP^a^ (mm Hg), mean (SD)			116.37 (13.80)		116.07 (14.53)		116.01 (13.09)		116.98 (15.19)		116.41 (12.36)		.95	
24-h DBP^b^ (mm Hg), mean (SD)			73.88 (9.12)		73.88 (9.58)		73.89 (8.88)		73.79 (9.63)		73.96 (8.46)		>.99	
24-h HR^c^ (bpm^d^), mean (SD)			71.43 (8.71)		73.96 (7.63)		71.45 (9.60)		72.00 (8.34)		68.33 (8.32)		<.001	
Total cholesterol (mmol/L), mean (SD)			5.44 (0.93)		5.40 (0.88)		5.38 (0.85)		5.42 (0.83)		5.57 (1.06)		.42	
LDL^e^ (mmol/L), mean (SD)			3.40 (0.82)		3.40 (0.77)		3.27 (0.86)		3.357 (0.71)		3.57 (0.92)		.06	
HDL^f^ (mmol/L), mean (SD)			1.493 (0.31)		1.44 (0.29)		1.47 (0.32)		1.55 (0.35)		1.49 (0.30)		.11	
Triglycerides (mmol/L), mean (SD)			1.19 (0.70)		1.2464762 (0.75)		1.2806604 (0.75)		1.12 (0.60)		1.13 (0.70)		.26	
Glucose (mmol/L), mean (SD)			5.28 (0.75)		5.34 (1.10)		5.34 (0.55)		5.25 (0.48)		5.21 (0.71)		.51	
BMI (kg/m^2^), mean (SD)			23.49 (3.49)		24.05 (4.40)		23.42 (3.2)		23.24 (3.54)		23.26 (2.56)		.30	
Log(CAC^g^+1), mean (SD)			1.16 (2.01)		0.97 (1.89)		1.08 (2.03)		1.13 (1.91)		1.45 (2.18)		.35	
Indexed RVEDV^h^ (mL/m^2^), mean (SD)			2087.94 (362.35)		1940.40 (273.89)		2046.38 (375.31)		2115.68 (339.49)		2249.46 (384.74)		<.001	
Indexed RVESV^i^ (mL/m^2^), mean (SD)			891.14 (256.14)		777.37 (188.92)		869.63 (279.37)		918.49 (231.37)		999.50 (267.26)		<.001	
Indexed LVEDV^j^ (mL/m^2^), mean (SD)			1994.67 (288.64)		1889.03 (238.83)		1953.58 (284.17)		2040.58 (277.55)		2092.64 (309.52)		<.001	
Indexed LVESV^k^ (mL/m^2^), mean (SD)			767.64 (193.41)		698.47 (168.88)		744.28 (192.64)		793.29 (182.67)		832.72 (203.37)		<.001	
LV^l^ mass (g/m^2^), mean (SD)			1227.55 (252.72)		1164.41 (221.55)		1199.44 (246.78)		1262.25 (264.28)		1281.77 (260.99)		.002	

^a^SBP: systolic blood pressure.

^b^DBP: diastolic blood pressure.

^c^HR: heart rate.

^d^bpm: beats per minute.

^e^LDL: low-density lipoprotein.

^f^HDL: high-density lipoprotein.

^g^CAC: coronary artery calcium.

^h^RVEDV: right ventricular end diastolic volume.

^i^RVESV: right ventricular end systolic volume.

^j^LVEDV: left ventricular end diastolic volume.

^k^LVESV: left ventricular end systolic volume.

^l^LV: left ventricle.

### Exploratory Factor Analysis

Using exploratory factor analysis with a cutoff of eigenvalue>1, we identified 3 latent factors measured via the Fitbit metrics on physical activity. The 3 factors identified were elevated METs, total activity, and others. While elevated METs represent the time engaged in moderate- and higher-level physical activity and catabolism measured in calories, total activity describes the average activity, including activity during nonexercise time, throughout the day.

Under factor 1, elevated METs, items with loadings of >0.4 included calories burned per day; minutes per day spent fairly active, defined as 3 to 6 METs; minutes per day spent very active, defined as ≥6 METs; and activity calories. For factor 2, total activity, items with loadings of >0.4 included number of steps per day, distance in kilometers per day, and number of floors per day. Factor 3 (others) items with loadings of >0.4 included sedentary time minutes and minutes spent lightly active, defined as 1.5 to 3 METs, where the loading value for sedentary time minutes was negative (Table 2).

**Figure 1:**
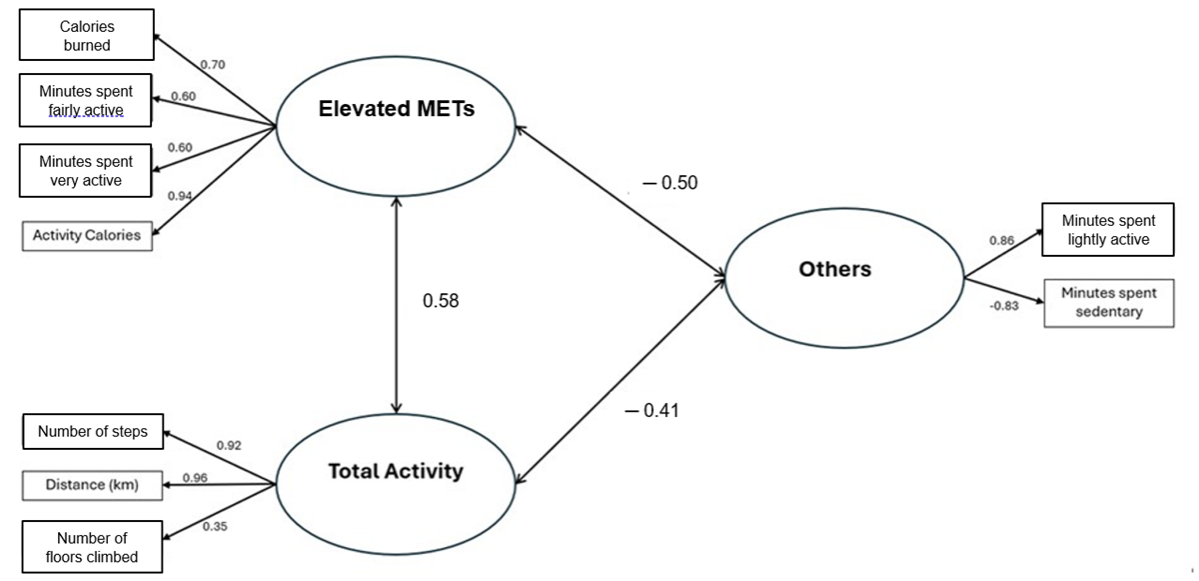
Confirmatory factor analysis and sequential equation modeling of the 9 Fitbit metrics. The values show the strength of association between the metrics and the factor and between the different factors. MET: metabolic equivalent of task.

Structural equation modeling was conducted on items and constructs, and the results are shown in Figure 1. There was a strong correlation (>0.6) between elevated METs and total activity.

**Table 2:** Item-to-scale (latent factor) correlation and Cronbach α of items.

			Factor loading		Item-to-scale correlation		Cronbach α
**Factor 1: elevated MET^a^ density^b^**							
	Calories burned per day	0.8394		0.7789		0.7937	
	Minutes spent fairly active per day	0.7769		0.7911		0.7849	
	Minutes spent very active per day	0.7511		0.7856		0.7888	
	Activity calories per day	0.7452		0.8683		0.7234	
**Factor 2: total activity^c^**							
	Number of steps per day	0.7061		0.8966		0.5589	
	Distance (km) per day	0.6916		0.9105		0.5217	
	Number of floors per day	0.8602		0.6956		0.9423	
**Factor 3: other nonsedentary time^d^**							
	Sedentary time per day	−0.8592		—^e^		—	
	Minutes spent lightly active per day	0.8896		—		—	

^a^MET: metabolic equivalent of task.

^b^Overall Cronbach α=0.8202.

^c^Overall Cronbach α=0.7815.

^d^Overall Cronbach α=0.7452.

^e^Not applicable.

### Reliability

For assessing internal consistency reliability, the Cronbach α exceeded 0.70 for all 3 constructs (elevated METs=0.820; total activity=0.781; others=0.745). Correlations between the factors and hypothesized items are shown in Table 2.

### Construct Validity

The correlation between self-reported PAI [36] and elevated METs and total activity was significant but weak (0.23 and 0.26, respectively; *P*<.001). The *others* factor was not correlated with PAI (*P*=.64).

### Regression Analysis

As there was a strong correlation between elevated METs and total activity, we included and tested the interaction term of elevated METs and total activity in the multivariate regression analysis.

### Lipid and Glucose Profiles and BMI

**Figure 2:**
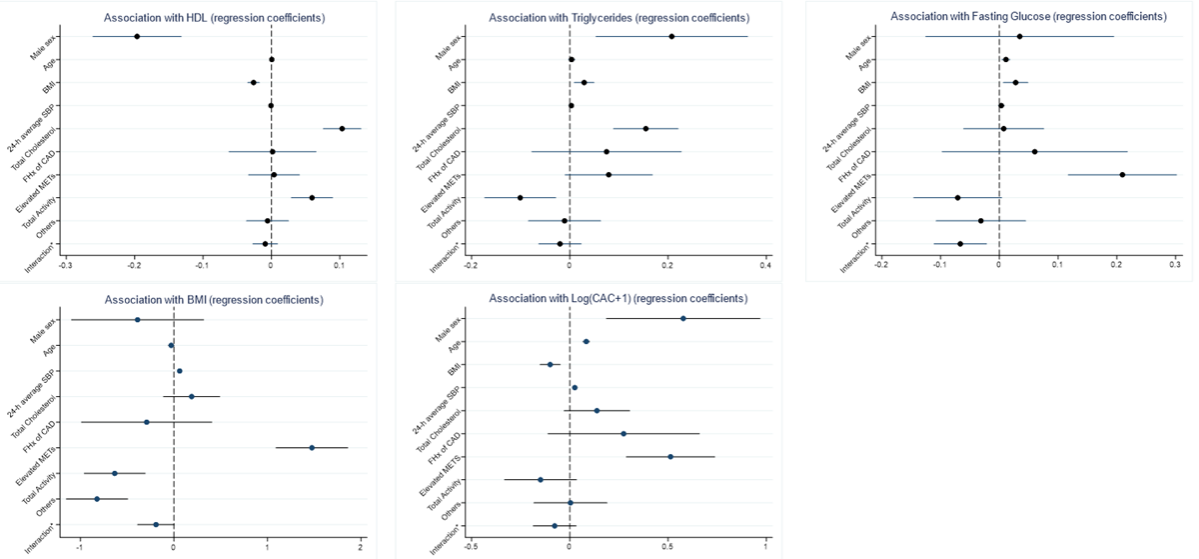
Regression coefficient plot of activity metric latent factors against lipid and glucose profiles, BMI and log(coronary artery calcium+1) adjusting for demographics and risk factors. CAC: coronary artery calcium; CAD: coronary artery disease; FHx: family history; HDL: high-density lipoprotein; MET: metabolic equivalent of task; SBP: systolic blood pressure.

In contrast to the PAI, which did not show any significant trend regarding lipid and glucose profiles and BMI, total activity was associated with significantly higher high-density lipoproteins (HDLs; β=0.06; *P*<.001) and lower triglycerides (β=−0.10; *P*=.006); total activity trended toward being associated with lower fasting glucose, and the interaction term of elevated METs and total activity was significantly associated with lower fasting glucose levels (β=−0.07; *P*=.004). The total activity and other factors were significantly associated with lower BMI (β=−0.63 and −0.82, respectively; *P*<.001), whereas the elevated METs factor was associated with higher BMI (β=1.47; *P*<.001). There was no significant relationship between the various factors and low-density lipoprotein (LDL; Table 3). The coefficient plots of the variables against the lipid and glucose profiles and BMI are shown in Figure 2.

**Table 3:** Linear regression of lipid and glucose profiles and BMI against demographics, clinical variables, and activity metrics.

	HDL^a^ (mmol/L)^b^				Triglycerides (mmol/L)^c^				LDL^d^ (mmol/L)^e^				Glucose (mmol/L)^f^				BMI (kg/m^2^)^g^		
	β (SE)	*t* test (*df*)	*P* value	β (SE)		*t* test (*df*)	*P* value	β (SE)		*t* test (*df*)	*P* value	β (SE)		*t* test (*df*)	*P* value	β (SE)		*t* test (*df*)	*P* value
Male sex	−0.20 (0.03)	−5.97 (10)	<.001	0.21 (0.08)		2.64 (10)	.009	0.09 (0.04)		2.10 (10)	.04	0.03 (0.08)		0.43 (10)	.67	−0.39 (0.36)		−1.08 (10)	.28
Age (y)	0.00 (0.00)	0.37 (10)	.71	0.00 (0.00)		1.10 (10)	.27	0.00 (0.00)		−0.46 (10)	.65	0.01 (0.00)		3.17 (10)	.002	−0.03 (0.02)		−1.91 (10)	.06
BMI (kg/m^2^)	−0.03 (0.00)	−6.05 (10)	<.001	0.03 (0.01)		2.83 (10)	.005	0.01 (0.01)		2.46 (10)	.01	0.03 (0.01)		2.60 (10)	.01	—^h^		—	—
24-h SBP^i^	0.00 (0.00)	−0.62 (10)	.53	0.00 (0.00)		1.21 (10)	.23	0.00 (0.00)		−0.58 (10)	.56	0.00 (0.00)		1.27 (10)	.20	0.06 (0.01)		5.41 (10)	<.001
Total cholesterol (mmol/L)	0.10 (0.01)	7.37 (10)	<.001	0.15 (0.03)		4.62 (10)	<.001	0.81 (0.02)		42.59 (10)	<.001	0.01 (0.03)		0.21 (10)	.83	0.19 (0.15)		1.23 (10)	.22
Family history of CAD^j^	0.00 (0.03)	0.05 (10)	.96	0.07 (0.08)		0.97 (10)	.34	−0.01 (0.04)		−0.23 (10)	.82	0.06 (0.08)		0.75 (10)	.45	−0.29 (0.35)		−0.82 (10)	.41
Elevated METs^k^	0.00 (0.02)	0.20 (10)	.84	0.08 (0.05)		1.75 (10)	.08	−0.03 (0.03)		−1.15 (10)	.25	0.21 (0.05)		4.46 (10)	<.001	1.48 (0.20)		7.55 (10)	<.001
Total activity	0.06 (0.02)	3.85 (10)	<.001	−0.10 (0.04)		−2.74 (10)	.006	−0.02 (0.02)		−0.76 (10)	.45	−0.07 (0.04)		−1.86 (10)	.06	−0.63 (0.17)		−3.81 (10)	<.001
Others	−0.01 (0.02)	−0.36 (10)	.72	−0.01 (0.04)		−0.29 (10)	.78	0.01 (0.02)		0.43 (10)	.67	−0.03 (0.04)		−0.81 (10)	.42	−0.82 (0.17)		−4.93 (10)	<.001
Interaction term of elevated METs and total activity	−0.01 (0.01)	−0.98 (10)	.33	−0.02 (0.02)		−0.91 (10)	.36	0.01 (0.01)		1.01 (10)	.31	−0.07 (0.02)		−2.92 (10)	.004	−0.19 (0.10)		−1.91 (10)	.06
_cons (constant of the model)	1.68 (0.16)	10.60 (10)	<.001	−0.98 (0.38)		−2.58 (10)	.01	−1.22 (0.21)		−5.71 (10)	<.001	3.63 (0.39)		9.25 (10)	<.001	17.22 (1.53)		11.22 (10)	<.001

^a^HDL: high-density lipoprotein.

^b^Adjusted *R*^2^=0.294.

^c^Adjusted *R*^2^=0.164.

^d^LDL: low-density lipoprotein.

^e^Adjusted *R*^2^=0.807.

^f^Adjusted *R*^2^=0.159.

^g^Adjusted *R*^2^=0.263.

^h^Not applicable.

^i^SBP: systolic blood pressure.

^j^CAD: coronary artery disease.

^k^MET: metabolic equivalent of task.

### Cardiac MRI and Coronary Calcium Score

**Figure 3:**
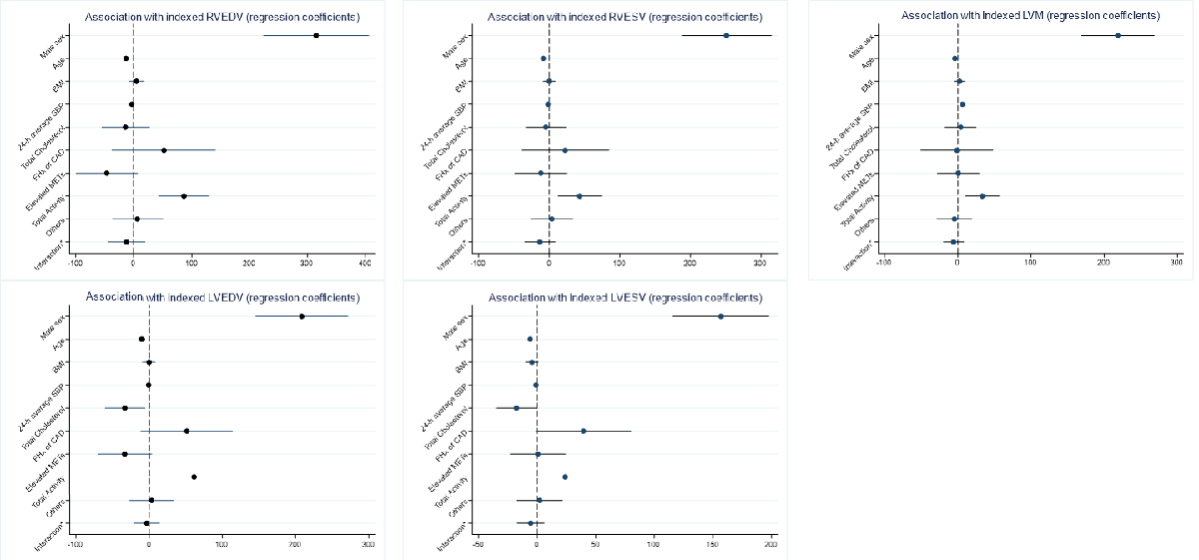
Regression coefficient plot of activity metric latent factors against cardiac magnetic resonance imaging dimensions adjusting for demographics and risk factors. CAD: coronary artery disease; FHx: family history; LVEDV: left ventricular end diastolic volume; LVESV: left ventricular end systolic volume; LVM: left ventricular mass; MET: metabolic equivalent of task; RVEDV: right ventricular end diastolic volume; RVESV: right ventricular end systolic volume; SBP: systolic blood pressure.

The elevated METs factor was significantly associated with higher log(CAC+1) (β=−0.51; *P*<.001; Table S1 in Multimedia Appendix 1). Total activity was significantly associated with higher indexed biventricular diastolic and systolic volumes and higher indexed left ventricular mass (Table 4). The coefficient plots of the variables against the log(CAC+1) and cardiac MRI variables are shown in Figures 2 and 3, respectively.

**Table 4:** Linear regression of cardiac magnetic resonance imaging dimensions against demographics, clinical variables, and activity metrics.

	Indexed RVEDV^a,b^				Indexed RVESV^c,d^				Indexed LVEDV^e,f^				Indexed LVESV^g,h^				Indexed LVM^i,j^		
	β (SE)	*t* test (*df*)	*P* value	β (SE)		*t* test (*df*)	*P* value	β (SE)		*t* test (*df*)	*P* value	β (SE)		*t* test (*df*)	*P* value	β (SE)		*t* test (*df*)	*P* value
Male sex	314.80 (46.29)	6.80 (10)	<.001	251.07 (32.26)		7.78 (10)	<.001	208.45 (32.06)		6.50 (10)	<.001	156.81 (20.88)		7.51 (10)	<.001	218.37 (25.34)		8.62 (10)	<.001
Age (y)	−12.73 (2.02)	−6.29 (10)	<.001	−8.53 (1.41)		−6.05 (10)	<.001	−9.78 (1.38)		−7.09 (10)	<.001	−5.94 (0.90)		−6.61 (10)	<.001	−4.12 (1.10)		−3.74 (10)	<.001
BMI (kg/m^2^)	5.32 (6.16)	0.87 (10)	.39	0.02 (4.29)		0.00 (10)	>.99	−0.42 (4.20)		−0.10 (10)	.92	−4.50 (2.73)		−1.65 (10)	.10	2.28 (3.31)		0.69 (10)	.49
24-h SBP^k^	−2.79 (1.53)	−1.82 (10)	.07	−1.98 (1.07)		−1.86 (10)	.06	−1.07 (1.03)		−1.04 (10)	.30	−1.00 (0.67)		−1.50 (10)	.14	6.25 (0.82)		7.67 (10)	<.001
Total cholesterol (mmol/L)	−13.91 (20.70)	−0.67 (10)	.50	−4.81 (14.42)		−0.33 (10)	.74	−32.76 (13.65)		−2.40 (10)	.02	−17.70 (8.89)		−1.99 (10)	.047	3.77 (10.80)		0.35 (10)	.73
Family history of CAD^l^	51.70 (45.16)	1.14 (10)	.25	22.65 (31.47)		0.72 (10)	.47	51.31 (31.65)		1.62 (10)	.11	39.50 (20.61)		1.92 (10)	.06	−1.38 (25.09)		−0.06 (10)	.96
Elevated METs^m^	−46.04 (27.09)	−1.70 (10)	.09	−11.82 (18.88)		−0.63 (10)	.53	−32.96 (18.46)		−1.79 (10)	.08	0.52 (12.02)		0.04 (10)	.97	0.53 (14.58)		0.04 (10)	.97
Total activity	86.23 (22.27)	3.87 (10)	<.001	43.23 (15.52)		2.79 (10)	.006	61.14 (14.99)		4.08 (10)	<.001	23.97 (9.76)		2.46 (10)	.01	33.25 (11.82)		2.81 (10)	.005
Others	6.79 (21.93)	0.31 (10)	.76	3.61 (15.28)		0.24 (10)	.81	3.48 (15.23)		0.23 (10)	.82	2.16 (9.92)		0.22 (10)	.83	−4.71 (12.18)		−0.39 (10)	.70
Interaction term of elevated METs and total activity	−11.83 (15.78)	−0.75 (10)	.45	−13.12 (11.00)		−1.19 (10)	.23	−3.31 (8.94)		−0.37 (10)	.71	−5.47 (5.82)		−0.94 (10)	.35	−5.84 (7.05)		−0.83 (10)	.41
_cons (constant of the model)	2844.39 (226.35)	12.57 (10)	<.001	1455.21 (157.73)		9.23 (10)	<.001	2702.53 (154.30)		17.51 (10)	<.001	1312.02 (100.47)		13.06 (10)	<.001	548.18 (122.74)		4.47 (10)	<.001

^a^RVEDV: right ventricular end diastolic volume.

^b^Adjusted *R*^2^=0.268.

^c^RVESV: right ventricular end systolic volume.

^d^Adjusted *R*^2^=0.286.

^e^LVEDV: left ventricular end diastolic volume.

^f^Adjusted *R*^2^=0.225.

^g^LVESV: left ventricular end systolic volume.

^h^Adjusted *R*^2^=0.250.

^i^LVM: left ventricular mass.

^j^Adjusted *R*^2^=0.385.

^k^SBP: systolic blood pressure.

^l^CAD: coronary artery disease.

^m^MET: metabolic equivalent of task.

### Quantification of Change in Lipid Profile and BMI per 1000 Increase in Steps

As the most used metric in total activity was step count, we sought to quantify the change in lipid profile and BMI with every 1000-step change. After adjusting for demographics and risk factors, every 1000-step increase per day increased HDL by 0.01 mmol/L, decreased triglycerides by 0.02 mmol/L, and reduced BMI by 0.11 kg/m^2^ (Table S2 in Multimedia Appendix 1).

## Discussion

### Principal Findings

The purpose of this study was to identify the underlying factors measured through an activity tracker, the Fitbit Charge HR, and extrapolate these factors to cross-sectional cardiovascular phenotypes. There has been no previous study to our knowledge conducted for this. Understanding the underlying factors measured by an activity tracker is important to set specific activity goals and prescribe precise physical activity domains using activity trackers. Previous studies have looked at the effectiveness of activity trackers in increasing physical activity [37] and largely focused on step counts in reduction of all-cause mortality [8,38].

This study revealed three factors measured by the Fitbit Charge HR: (1) elevated METs (calories burned per day, minutes per day spent fairly active, 3-6 METs; minutes per day spent very active, ≥6 METs; and activity calories), (2) total activity (number of steps per day, distance in kilometers per day, and number of floors per day), and (3) others (sedentary time minutes and minutes spent lightly active, 1.5-3 METs).

Total activity, represented by steps, distance, and floors, appeared to be consistently associated with a favorable cardiovascular risk profile in our study. It appears that the absolute volume of activity, quantified via total activity metrics, was more consistent in terms of positive cardiovascular benefit [39-41] compared to elevated METs. There was a dose-dependent increase in HDL and a reduction in triglycerides and BMI. HDL is more sensitive to exercise compared to LDL and triglycerides [42]. The lack of decreased LDL with total activity may be due to the study being observational rather than interventional in nature, where an exercise program was shown to reduce LDL, especially when the mean LDL of the participants was normal at 3.41 (SD 0.83) mmol/dL. Given that the adjusted *R*^2^ of the model was high at >0.80, it might also be that other factors played a much larger role in influencing LDL levels compared to physical activity. Compared to self-reported PAI, which had no association with lipid and glucose profiles and BMI, these results suggest that wearable-quantified activity metrics are more accurate and precise in recording activity level, as evidenced by significant associations with cross-sectional cardiometabolic profiles.

There was also significant cardiovascular remodeling based on total activity and self-reported PAI, with significantly larger indexed biventricular systolic and diastolic volumes and indexed left ventricular mass after adjustment for baseline demographics and risk factors. This finding was also consistent with those of previous exercise studies [43,44]. In our study, we can only conclude that total activity was associated with physiological adaptation of the heart, but the functional consequence is unknown.

Regarding the association between elevated METs and higher log(CAC+1), one possible hypothesis is the reverse J curve of exercise [45,46], where excessive exercise gradually precipitates cardiovascular damage. A UK Biobank study demonstrated that the cardiovascular protective effect was lost in the 75th percentile of moderate to vigorous physical activity and above [47], and a systematic review showed that endurance training increased the odds of having high CAC but without increased prevalence of high-risk plaque [48]. Another possible explanation is a change in participant behavior due to the Hawthorne effect [49]. The METs calculated via Fitbit were based on heart rate and step count over a prespecified period [50]; it is possible that less healthy participants attempted to put in extra exercise time over the 7-day observation period, and with poorer heart rate recovery [51], this would increase their scores in elevated METs. Total activity detailed the average step count and distance covered daily and was harder to inflate compared to elevated METs. Hence, this finding warrants further prospective observational studies or longer periods of monitoring before drawing conclusions.

There are also very few studies that have correlated step count and activity levels to reduced subclinical atherosclerosis [52]. However, there is consistent literature suggesting that increasing step count reduces mortality and cardiovascular events [8,38,53]. Previous population studies have suggested that exercise is a risk modifier, especially in patients with high CAC [54]. As shown in our study, this could be mediated through increased HDL and reduced triglycerides, BMI, and fasting glucose [55,56]. While the pathophysiology might be hard to prove in human studies, animal studies have shown that exercise training reduces glucose and triglyceride levels in LDL-knockout mice [57] and reduces hyperlipidemia-induced cardiac damage through reducing oxidative stress and inflammation in apolipoprotein E–deficient mice [58], hence reducing mortality in atherosclerosis-predisposed animals. HDL is an antioxidant that provides potent protection to LDL from oxidative damage by free radicals, hence inhibiting the generation of proinflammatory oxidized lipids [59], and aerobic exercise has been found to increase both HDL quantity and quality [60]. This might be a reason for the lack of significant association between total activity and presence of atherosclerosis quantified through log(CAC+1) as atherosclerosis might have been caused by other genetic and epigenetic factors and physical activity is but a risk modifier.

Our study showed that there are distinct characteristics conveyed by the different Fitbit activity metrics. Interpreting them individually may not be as helpful; there is a need to recognize the underlying factor described by each metric to make more precise recommendations for exercise and physical health. This study shows that increased activity regardless of MET intensity, represented by total activity, has significant beneficial associations with cardiometabolic profiles. After accounting for gender, BP, and other risk factors, every 1000-step increase per day increased HDL by 0.01 mmol/L, reduced triglycerides by 0.02 mmol/L, and reduced BMI by 0.11 kg/m^2^. From a clinical perspective, it may be reasonable to look at the metrics represented sequentially and hierarchically, first ensuring that the individual has achieved reasonable total activity before focusing on the intensity of the activity quantified through the metrics in the elevated METs factor. For example, for patients with no CAC, physicians can recommend 10,000 steps per day [61] and no restriction on activity; conversely, for patients with high CAC who are looking for exercise recommendations, focusing mainly on 10,000 steps per day and moderating very vigorous activity based on effort limitation, especially if the patient had no exercise regime before, may be reasonable.

This was an observational study and not an interventional study, hence limiting it to associations. The 7-day observation period, due to limitations in device availability, may also not be a true representation of activity levels in all patients, although the study team counseled participants to continue their usual activity levels. In addition, this study comprised mainly an Asian population; hence, the generalizability of these findings to other populations may be limited. It will be helpful to use the same framework and explore these associations in other populations. The upcoming 10-year follow-up for this prospective cohort will also shed light on the impact of wearable-quantified physical activity on cardiovascular events stratified by baseline CAC scores.

### Conclusions

There are different characteristics conveyed by the different Fitbit physical activity metrics. This study yielded 3 main latent factors and described their associations with cardiovascular health. Total activity, rather than elevated METs, was associated with beneficial effects on cardiometabolic profiles. Total activity appears to be more sensitive and precise than self-reported PAI, with significant associations with lipid and glucose profiles and BMI. One can consider interpreting activity metrics on wearables sequentially and hierarchically, focusing first on metrics in the total activity factor before considering metrics in the elevated METs factor when providing physical activity advice.

## Appendix

Multimedia Appendix 1 Supplementary tables.

## Abbreviations

BP: blood pressure
CAC: coronary artery calcium
HDL: high-density lipoprotein
LDL: low-density lipoprotein
MET: metabolic equivalent of task
MRI: magnetic resonance imaging
PAI: physical activity index

## Data Availability

The datasets generated or analyzed during this study are not publicly available as recruitment is still ongoing and the participants have not completed their follow-up but are available from the corresponding author on reasonable request.
