# Supplementary material for: Relationship Between Activity Tracker Metrics and the Physical Activity Index and Their Association With Cardiometabolic Phenotypes, Subclinical Atherosclerosis, and Cardiac Remodeling: Cross-Sectional Study
Source: JMIR Mhealth Uhealth. 2025 Sep 24;13:e71213. doi: 10.2196/71213 (PMC12508664; doi:10.2196/71213)
Supplement: Multimedia Appendix 1 [file mhealth_v13i1e71213_app1.docx]

| Supplementary Table 1: Linear Regression of Log(CAC+1) against demographics, clinical variables and Activity Metrics | | | | | | |
| --- | --- | --- | --- | --- | --- | --- |
| Log(CAC+1) | Coefficient | Std. err. | t | P>t | [95% conf. interval] | |
|  |  |  |  |  |  |  |
| Male | 0.58 | 0.20 | 2.90 | 0.004 | 0.19 | 0.97 |
| Age (years) | 0.08 | 0.01 | 9.73 | 0.000 | 0.07 | 0.10 |
| BMI (kg/m2) | -0.10 | 0.03 | -3.83 | 0.000 | -0.15 | -0.05 |
| 24hr SBP | 0.03 | 0.01 | 3.97 | 0.000 | 0.01 | 0.04 |
| Total Cholesterol (mmol/L) | 0.14 | 0.08 | 1.62 | 0.105 | -0.03 | 0.30 |
| Family history of coronary artery disease | 0.27 | 0.20 | 1.40 | 0.163 | -0.11 | 0.66 |
| Elevated METs | 0.51 | 0.11 | 4.47 | 0.000 | 0.29 | 0.74 |
| Total Activity | -0.15 | 0.09 | -1.60 | 0.111 | -0.33 | 0.03 |
| Others | 0.00 | 0.09 | 0.04 | 0.972 | -0.18 | 0.19 |
| Interaction* | -0.08 | 0.06 | -1.39 | 0.167 | -0.19 | 0.03 |
| _cons | -4.66 | 0.96 | -4.85 | 0.000 | -6.54 | -2.77 |
| Adjusted R2 | | | | | 0.337 | |
| *CAC, coronary artery calcium; BMI, body mass index; SBP, systolic blood pressure; METs, metabolic equivalent tasks; Interaction*, interaction term between Elevated METs and Total Activity* | | | | | | |

| Supplementary Table 2: Linear Regression of Lipid profile and BMI against demographics, clinical variables and every 1000 steps | | | | | | | | | | | | |
| --- | --- | --- | --- | --- | --- | --- | --- | --- | --- | --- | --- | --- |
|  | HDL (mmol/L) | | | | Triglycerides (mmol/L) | | | | BMI (kg/m2) | | | |
|  | β | SE | t | p | β | SE | t | p | β | SE | t | p |
| Male | -0.18 | 0.03 | -6.49 | 0.000 | 0.24 | 0.07 | 3.67 | 0.000 | 1.03 | 0.31 | 3.29 | 0.001 |
| Age (years) | 0.00 | 0.00 | 0.15 | 0.880 | 0.00 | 0.00 | 1.23 | 0.219 | -0.04 | 0.02 | -2.66 | 0.008 |
| BMI (kg/m2) | -0.03 | 0.00 | -6.49 | 0.000 | 0.04 | 0.01 | 3.74 | 0.000 |  |  |  |  |
| 24hr SBP | 0.00 | 0.00 | -0.73 | 0.466 | 0.00 | 0.00 | 1.63 | 0.104 | 0.08 | 0.01 | 7.07 | 0.000 |
| Total Cholesterol (mmol/L) | 0.10 | 0.01 | 7.34 | 0.000 | 0.16 | 0.03 | 4.65 | 0.000 | 0.21 | 0.16 | 1.26 | 0.209 |
| Family history of coronary artery disease | 0.00 | 0.03 | -0.06 | 0.956 | 0.07 | 0.08 | 0.96 | 0.337 | -0.36 | 0.38 | -0.95 | 0.343 |
| Per 1000 steps | 0.01 | 0.00 | 3.30 | 0.001 | -0.02 | 0.01 | -2.28 | 0.023 | -0.11 | 0.05 | -2.36 | 0.019 |
| _cons | 1.58 | 0.15 | 10.27 | 0.000 | -1.10 | 0.37 | -3.01 | 0.003 | 15.67 | 1.63 | 9.64 | 0.000 |
|  | Adjusted R2 | | 0.288 | | Adjusted R2 | | 0.159 | | Adjusted R2 | | 0.161 | |
| *HDL, high density lipoprotein; BMI, body mass index; β, linear regression coefficient; SE, standard error; p, p-value; SBP, systolic blood pressure; CAD, coronary artery disease* | | | | | | | | | | | | |

| Table 3: Linear Regression of Lipid, glucose profile and BMI against demographics, clinical variables and Activity Metrics | | | | | | | | | | | | | | | | | | | | |
| --- | --- | --- | --- | --- | --- | --- | --- | --- | --- | --- | --- | --- | --- | --- | --- | --- | --- | --- | --- | --- |
|  | HDL (mmol/L) | | | | Triglycerides (mmol/L) | | | | LDL (mmol/L) | | | | Glucose (mmol/L) | | | | BMI (kg/m2) | | | |
|  | β | SE | t | p | β | SE | t | p | β | SE | t | p | β | SE | t | p | β | SE | t | p |
| Male | -0.20 | 0.03 | -5.97 | 0.000 | 0.21 | 0.08 | 2.64 | 0.009 | 0.09 | 0.04 | 2.10 | 0.036 | 0.03 | 0.08 | 0.43 | 0.671 | -0.39 | 0.36 | -1.08 | 0.280 |
| Age (years) | 0.00 | 0.00 | 0.37 | 0.713 | 0.00 | 0.00 | 1.10 | 0.270 | 0.00 | 0.00 | -0.46 | 0.648 | 0.01 | 0.00 | 3.17 | 0.002 | -0.03 | 0.02 | -1.91 | 0.057 |
| BMI (kg/m2) | -0.03 | 0.00 | -6.05 | 0.000 | 0.03 | 0.01 | 2.83 | 0.005 | 0.01 | 0.01 | 2.46 | 0.014 | 0.03 | 0.01 | 2.60 | 0.010 | - | - | - | - |
| 24hr SBP | 0.00 | 0.00 | -0.62 | 0.534 | 0.00 | 0.00 | 1.21 | 0.225 | 0.00 | 0.00 | -0.58 | 0.562 | 0.00 | 0.00 | 1.27 | 0.204 | 0.06 | 0.01 | 5.41 | 0.000 |
| Total Cholesterol (mmol/L) | 0.10 | 0.01 | 7.37 | 0.000 | 0.15 | 0.03 | 4.62 | 0.000 | 0.81 | 0.02 | 42.59 | 0.000 | 0.01 | 0.03 | 0.21 | 0.831 | 0.19 | 0.15 | 1.23 | 0.218 |
| Family history of CAD | 0.00 | 0.03 | 0.05 | 0.958 | 0.07 | 0.08 | 0.97 | 0.335 | -0.01 | 0.04 | -0.23 | 0.818 | 0.06 | 0.08 | 0.75 | 0.454 | -0.29 | 0.35 | -0.82 | 0.411 |
| Elevated METs | 0.00 | 0.02 | 0.20 | 0.841 | 0.08 | 0.05 | 1.75 | 0.081 | -0.03 | 0.03 | -1.15 | 0.249 | 0.21 | 0.05 | 4.46 | 0.000 | 1.48 | 0.20 | 7.55 | 0.000 |
| Total Activity | 0.06 | 0.02 | 3.85 | 0.000 | -0.10 | 0.04 | -2.74 | 0.006 | -0.02 | 0.02 | -0.76 | 0.446 | -0.07 | 0.04 | -1.86 | 0.064 | -0.63 | 0.17 | -3.81 | 0.000 |
| Others | -0.01 | 0.02 | -0.36 | 0.717 | -0.01 | 0.04 | -0.29 | 0.775 | 0.01 | 0.02 | 0.43 | 0.668 | -0.03 | 0.04 | -0.81 | 0.418 | -0.82 | 0.17 | -4.93 | 0.000 |
| Interaction* | -0.01 | 0.01 | -0.98 | 0.326 | -0.02 | 0.02 | -0.91 | 0.363 | 0.01 | 0.01 | 1.01 | 0.314 | -0.07 | 0.02 | -2.92 | 0.004 | -0.19 | 0.10 | -1.91 | 0.057 |
| _cons | 1.68 | 0.16 | 10.60 | 0.000 | -0.98 | 0.38 | -2.58 | 0.010 | -1.22 | 0.21 | -5.71 | 0.000 | 3.63 | 0.39 | 9.25 | 0.000 | 17.22 | 1.53 | 11.22 | 0.000 |
|  | Adjusted R2 | | 0.294 | | Adjusted R2 | | 0.164 | | Adjusted R2 | | 0.807 | | Adjusted R2 | | 0.159 | | Adjusted R2 | | 0.263 | |
| *HDL, high density lipoprotein; LDL, low-density lipoprotein; BMI, body mass index; β, linear regression coefficient; SE, standard error; p, p-value; SBP, systolic blood pressure; CAD, coronary artery disease; METs, metabolic equivalent tasks; Interaction*, interaction term between Elevated METs and Total Activity* | | | | | | | | | | | | | | | | | | | | |
